# Electrochemical Performance of a Carbon Nanotube/La-Doped TiO_2_ Nanocomposite and its Use for Preparation of an Electrochemical Nicotinic Acid Sensor

**DOI:** 10.3390/s8117085

**Published:** 2008-11-07

**Authors:** Jing Wu, Hanxing Liu, Zhidong Lin

**Affiliations:** 1 State Key Laboratory of Advanced Technology for Materials Synthesis and Processing, Wuhan University of Technology, Wuhan, P.R. China, 430070; 2 School of Materials Science Engineering, Wuhan Institute of Technology, Wuhan, Hubei, P.R. China 430073. E-mail: zhidonglin@yahoo.com.cn (Z.D.L.)

**Keywords:** Carbon nanotube/La-TiO_2_ nanocomposite (CLTN), sensor, nicotinic acid (NA)

## Abstract

A carbon nanotube/La-doped TiO_2_ (La-TiO_2_) nanocomposite (CLTN) was prepared by a procedure similar to a complex/adsorption process. Scanning electron microscopy (SEM) images show that the La-TiO_2_ distributes on the carbon nanotube walls. The CLTN was mixed with paraffin to form a CLTN paste for the CLTN paste electrode (CLTNPE). The electrochemical characteristics of CLTNPE were compared with that of conventional carbon electrodes such as the carbon paste electrode (CPE) and glass carbon electrode (GC). The CLTNPE exhibits electrochemical activity and was used to investigate the electrochemistry of nicotinic acid (NA). The modified electrode has a strong electro-catalytic effect on the redox of NA. The cyclic voltammetry (CV) redox potential of NA at the CLTNPE is 320 mV. The oxidation process of NA on the CLTNPE is pH dependent. A sensitive chronoamperometric response for NA was obtained covering a linear range from 1.0×10^-6^ mol·L^-1^ to 1.2×10^-4^ mol·L^-1^, with a detection limit of 2.7×10^-7^ mol·L^-1^. The NA sensor displays a remarkable sensitivity and stability. The mean recovery of NA in the human urine is 101.8%, with a mean variation coefficient (RSD) of 2.6%.

## 1. Introduction

Carbon nanotubes (CNT) are one of the most important nanomaterials due to their unique electronic, metallic and structural characteristics [1]. They have been intensively used for electrocatalytic and sensing applications [2-5]. Most CNT-sensing research has focused on the ability of surface-confined CNTs to promote electron-transfer reactions with electroactive species. In recent years, composite materials based on CNTs have attracted great interest from researchers. Wang *et al* [6] synthesized carbon nanotube-conducting polymer composite nanowires. By combining the attractive properties of CNT and conducting polymers, the new nanocomposite opens up new opportunities, ranging from chemical sensors to molecular electronic devices. Zhang and Gorski [7] have reported a carbon nanotube-chitosan system, which has been successfully used to modify electrode surfaces for sensor and biosensor development. Metallic nanoparticles were attached to the CNT surface by electrodeposition to form new nanomaterials [8]. The sidewalls of nanotubes serve as both the electrodeposition template and as the wire electrically connecting the deposited nanoparticles. It has been reported that carbon nanotubes-cobalt hexacyanoferrate nanoparticles-chitosan film can be used to construct biosensors [9]. In our laboratory, we have successfully synthesized Fe-CNT nanocomposites using an exfoliation/adsorption process to intercalate Fe^3+^ into graphite oxide layers; the intercalated F^3+^ was reduced in a H_2_ atmosphere resulting Fe-CNT nanoparticles [10]. The Fe-CNT nanocomposite was used for a mediator-free glucose biosensor. There are few reports exploring the La-doped TiO_2_ nanoparticle (La-TiO_2_) composite with the CNT. The electrochemical characteristics and applications of this kind of materials in the field of analytical chemistry haven't been reported yet. In the present work, we synthesized a carbon nanotube/La-TiO_2_ nanocomposite (CLTN) and studied its electrochemical properties. The CLTN was used as an electrochemical sensing platform for the detection of vitamin pp.

**Figure 1.**
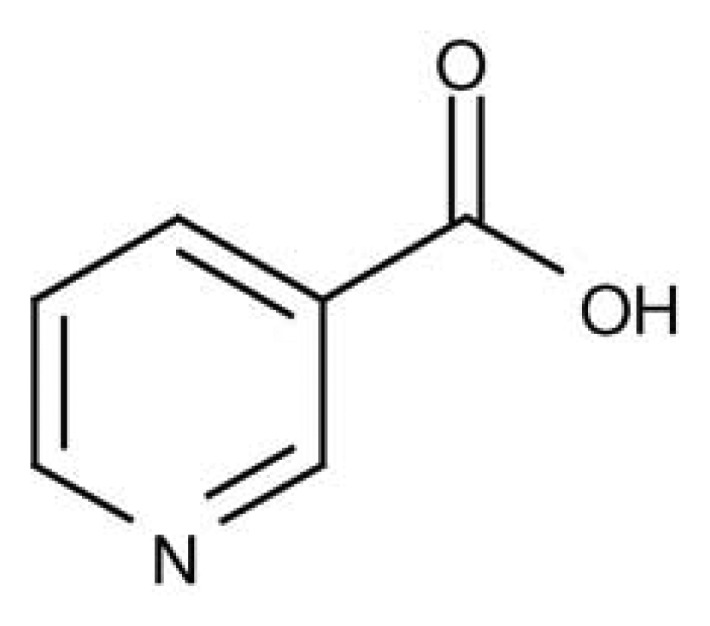


Vitamin pp, usually referred to as nicotinic acid (pyridine 3-carboxylic acid, NA), niacin or vitamin B_3_ is shown in Figure 1. It displays important biological activity and is is one of the important water soluble vitamins that is easily lost when boiled in water, and it can't be stored in the human body. NA is usually found in urine.

The analysis of NA has attracted considerable interest due to its anti-oxygen free radicals and anti-lipoperoxidation action [11], which can lower the concentrations of cholesterol and triglyceride in the blood [12-13].

Recently, various analytical methods have been used to detect NA, such as high performance liquid chromatography (HPLC) [14-15], HPLC/gas chromatography-mass spectrometry (GC-MS)[16], flow-injection spectrophotometric [17], micellar electrokinetic capillary chromatography [18-19], Fluorimetric determination [20-21], in-capillary enzyme reaction method [22], and luminescence [23]. Among the above methods, few reports have been investigated the electrochemistry of NA. Takayama *et al.* [24] have reported the bioelectrocatalytic hydroxylation of nicotinic acid with a carbon paste electrode modified with bacterial cells of *Pseudomonas fluorescens*. The electrochemical detection of NA was realized in the presence of electron transfer mediators.

In the present work, the complex/adsorption process was used to prepare carbon nanotube/La-TiO_2_ nanocomposite (CLTN) by compositing La-TiO_2_ onto CNTs. Scanning electron microscopy (SEM) was utilized to characterize the synthesized CLTN. The CLTN was used to prepare a CLTN paste electrode (CLTNPE) by mixing CLTN with paraffin. The electrochemical characteristics of CLTNPE were studied using K_3_Fe(CN)_6_ and ascorbic acid solutions, and the results were compared with those of conventional carbon electrodes, such as a carbon paste electrode (CPE) and a glass carbon electrode (GC). It was found that the electron transfer rate of CLTNPE is faster than that of conventional carbon electrodes. A well-defined oxidation peak of NA was obtained with the proposed CLTNPE, which was thus used to detect NA electrochemically. Satisfactory linear range and recovery results were obtained with human urine.

## 2. Results and Discussion

### 2.1. Characteristics of CLTN

**Figure 2.**
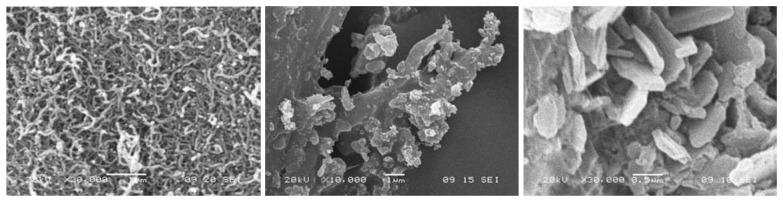
The images of SEM (a) CNT. (b) La-TiO2. (c) CLTN.

Figure 2 presents typical SEM images of La-TiO_2_ nanoparticles (a), CNT (b) and CLTN (c). It can be seen that the La-TiO2 nanoparticles have the typical dispersive and intermittent shape with diameters around 30 nm; some large ones reach about 50 nm. The diameter of CNTs in Figure 1 (b) is between 30 and 80 nm. Figure 1 (c) reveals that the La-TiO_2_ nanoparticles are tightly clinging to the o-CNTs, indicating the formation of CLTN. The above results suggested that the electron-deficient atoms of La and Ti in La-TiO_2_ nanoparticle easily complex with carboxylic groups of the o-CNTs, which is the synthetical basis of CLTN.

### 2.2. Electrochemical characteristics of CLTNPE

**Figure 3.**
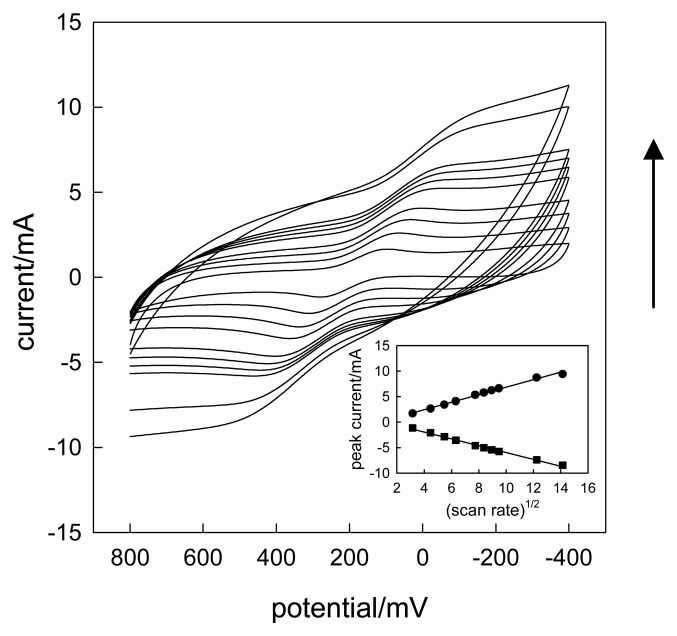
Cyclic voltammograms obtained with the CLTNPE in 0.1 mol·L^-1^ KCl containing 20 m mol·L^-1^ potassium ferricyanide (pH 7.0). From down to up, the scan rate is 10, 20, 30, 40, 60, 70, 80, 90, 150, 200 mV·s^-1^, respectively.

The electrochemical sensing characteristics of CLTNPE were investigated with typical redox species, such as potassium ferricyanide. Figure 3 displays the cyclic voltammograms of current recorded as function of scan rate shows a linear I_p_ *vs.* v^1/2^ relationship covering the 10 - 200 mV·s^-1^ range. This indicates that the current is controlled by a semi-infinite linear diffusion with the low scan rate of cyclic voltammetry (CV). The redox peak potentials shift slightly as the scan rate increases. We found that the redox peaks are not obvious while the scan rate is more than 90 mV·s^-1^. The results may be attributed, at least in part, to the slower increase rate of oxidation and reduction compared with that of scan rate.

In fact, the electrochemical oxidation of potassium ferricyanide was investigated at different concentrations, ranging from 0.01mmol·L^-1^ to 10 mmol·L^-1^, at CLTNPE. There is a linear relationship between the potassium ferricyanide concentration and oxidation current. These experimental results indicate that CLTNPE have good ability of electron-transfer and which can be used for quantitative determination.

**Figure 4.**
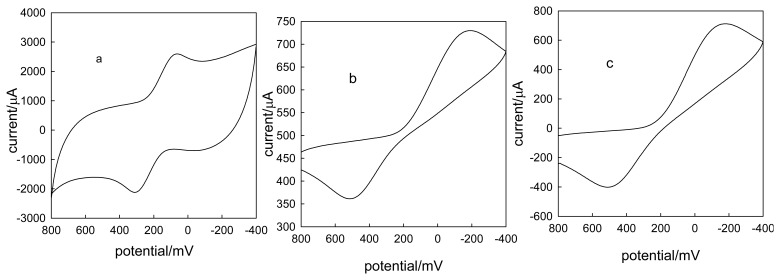
Cyclic voltammograms obtained with the CLTNPE (a), GC (b) and CPE (c) in 20 mmol L^-1^ potassium ferricyanide containing 0.1 mol·L^-1^ KCl (pH 7.0). Scan rate: 50 mV s^-1^

**Figure 5.**
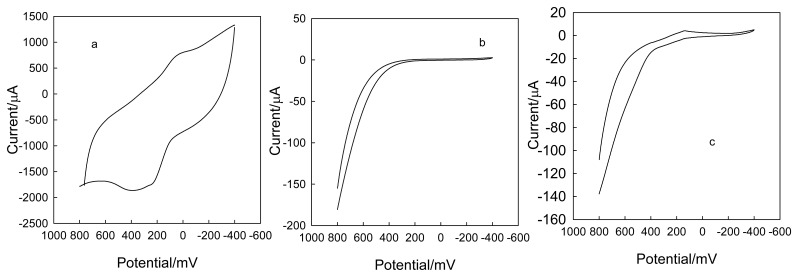
Cyclic voltammograms obtained with the CLTNPE (a), GC (b) and CPE (c) in 20 mmol L^-1^ ascorbic acid containing 0.1 mol L^-1^ KCl (pH 7.0) Scan rate: 50 mV s^-1^.

In order to further investigate the electrochemistry of CLTNPE, potassium ferricyanide and ascorbic acid were utilized and the results compared with those of conventional carbon electrodes, such as CPE and GC. Figure 4 shows the typical cyclic voltammograms of ferricyanide at 50 mV·s^-1^ on CLTNPE, GC and CPE without any pretreatment. The CLTNPE (Figure 4a) displays a couple of well-defined redox peaks with peak potential at 281.0 mV (Epa) and 99.0 mV (Epc). Compared with that obtained on GC (Figure 4b) and CPE (Figure 4c), the oxidation potential on the CLTNPE shifts negatively to 235.0 mV and 213.3 mV, respectively. The reduction peak potential on the CLTNPE shows the positive shifts of 290.8 mV and 300.6 mV. For ascorbic acid (Figure 5), there is no obvious redox response using the GC (Figure 5b) and CPE (Figure 5c) in the selected potential range, while an obvious quasi-reversible redox peaks were observed at CLTNPE (Figure 5a). The oxidation and reduction peak potentials are 400.0 mV and 37.6 mV, respectively. From above results, we conclude that CLTNPE can accelerate the electron-transfer of redox species and improve the reversibility of redox reaction.

### 2.3. Electrochemical characteristics of NA on CLTNPE

**Figure 6.**
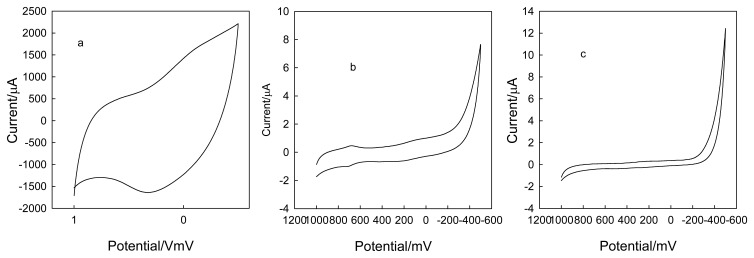
Cyclic voltammograms obtained with the CLTNPE (a), GC (b) and CPE (c) in 20 mmol L^-1^ NA containing 0.1 mol L^-1^ KCl (pH 10.0). Scan rate: 50 mV s^-1^.

Figure 6 shows the cyclic voltammograms obtained with the CLTNPE, GC, and CPE in 0.1 mol·L^-1^ KCl (pH 10) containing 20 mmol·L^-1^ NA. The couple of oxidation and reduction peaks potentials were found to be 715.6 mV and 683.0 mV, using GC as shown in Figure 6b. When using CLTNPE as the working electrode (Figure 6a), a sharp oxidation peak is apparent at 320.0 mV and shifts negatively 395.6 mV compared with that on the GC electrode. No apparent reduction peak was located at the cyclic voltammogram. Such a significant oxidation potential shift may be attributed to the fast electron transfer rate of CLTNPE. No redox peaks was observed in the cyclic voltammogram of CPE, indicating NA can not be oxidized and reduced on CPE (Figure 6c). Although the GC can realize redox process of NA, its high oxidation potential prevents its practical applications. Because ascorbic acid, uric acid and dopamine have a similar oxidation potential, they may interfere with NA detection in body fluid matrices, such as urine and blood. The low oxidation potential of NA on CLTNPE would avoid such kind of interferences. Additionally, the oxidation peak current of NA on CLTNPE is much higher than that on GC, indicating that the CLTNPE may be sued as sensitive tool for electrochemical detection of NA.

**Figure 7.**
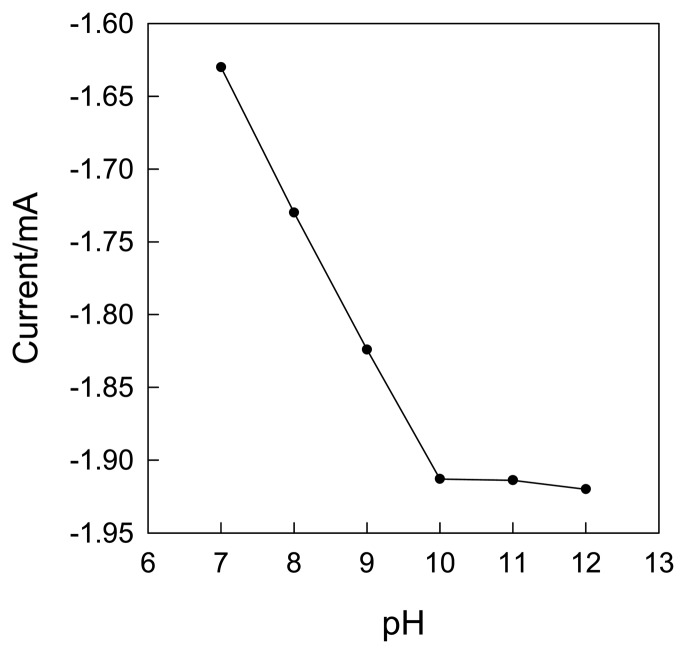
The effect of pH on NA oxid1zation current. NA: 10 mmol·L^-1^ containing 0.1 mol·L^-1^ KCl.

The NA is a typical kind of acid, which has very low solubility in acidic solutions, so neutral and alkaline solutions were used to investigate the pH effect. The pH dependence of CLTNPE over the pH range from 7 to 12 in 0.1 mol·L^-1^ KCl containing 10 mmol·L^-1^ NA is illustrated by cyclic voltammetry, as shown in Figure 7. It reveals that the response of current increases significantly from pH 7 to pH 10. Above the pH 10, the peak current tends to directly level off with the increase of pH. The CLTNPE has maximum sensitivity around pH 10. The results show that alkaline solutions favor the oxidation of NA on the CLTNPE.

The influence of potential scan rate on the oxidation and reduction peak current has been investigated by cycle voltammetry in the range of 30-90 mV·s^-1^. It reveals that the redox peak current is proportional to the square root of scan rate, indicating a diffusion confined redox process. And the redox peak potentials were slightly shifted with the scan rate increase. It was found that the redox peak current only varied slightly while the scan rate was more than 90 mV·s^-1^, which was likely attributed to the slow increase of oxidation rate.

**Figure 8.**
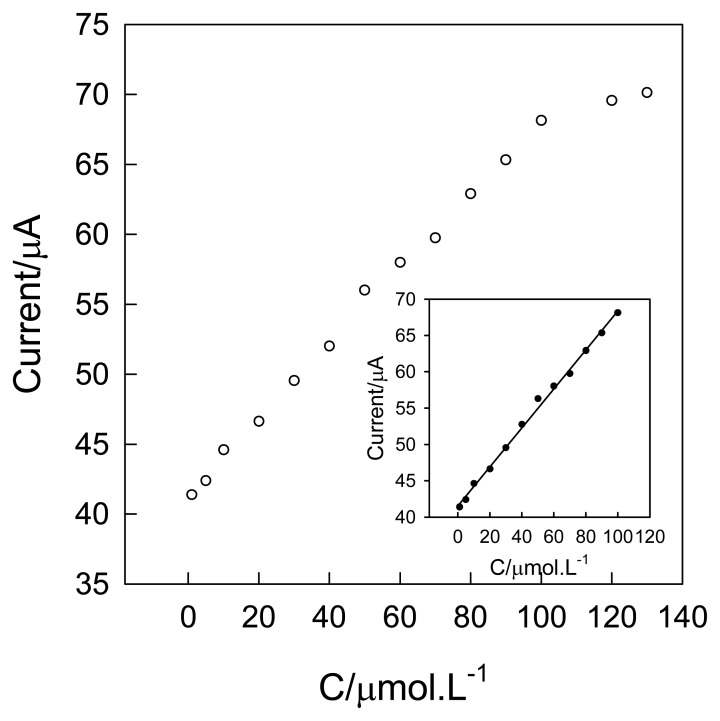
Steady state calibration curve of NA sensor in 0.1 mol L^-1^ KCl (pH 10.0) at oxidization potential of 320.0 mV (vs SCE).

Figure 8 displays the calibration curve for NA using chronoamperometry with the applied potential 320.0 mV in 0.1 mol·L^-1^ KCl (pH 10). A linear relationship between the chronoamperometric response and the concentration of NA was obtained covering the range from 1.0×10^-6^ molL^-1^ to 1.1×10^-4^ mol·L^-1^ with a detection limit of 2.7×10^-7^ mol·L^-1^. The linear regression equation being I (μA) = 0.2679C (μmol·L^-1^) + 41.55 with a correlation coefficient of 0.9963. The response is saturated at the NA concentration of 1.2×10^-4^ mol L^-1^.

The stability of the CLTNPE was investigated by chronoamperometry with a 0.1 mmol·L^-1^ solution of NA. The variation coefficient (RSD) was 1.9% for five successive assays in NA solution. RSD is 3.2 % for five interval assays in NA solution. The results showed that the CLTNPE as NA sensor has promising stability and reproducibility.

**Figure 9.**
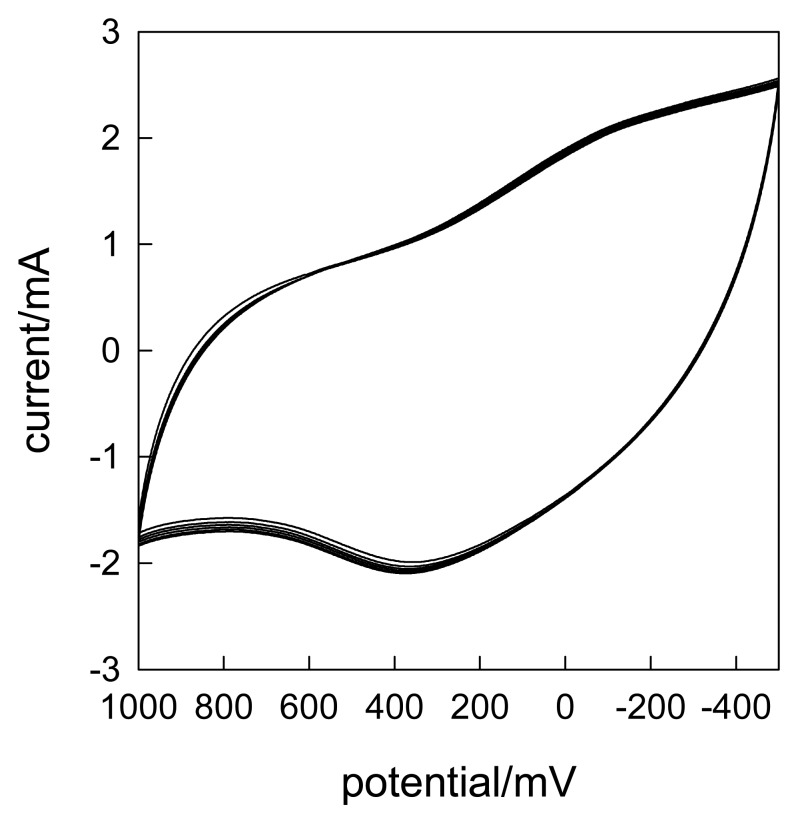
Eleven cyclic voltammograms obtained with the CLTNPE in 0.1 mmol.L^-1^ of NA. Scan rate: 50 mV/s

In order to further show the nice stability and reproducibility, the successive CVs were scanned in 0.1 mmol·L^-1^ of NA using the CLTNPE, as shown in Figure 9. It can be seen the peak current changes lightly. The RSD is 1.4% and the peak potential is nearly no shift. The results indicate that the proposed electrode has perfect stability and reproducibility.

Owing to the specific properties of CLTNPE with respect to NA, few reagents should interfere with the response of NA under the applied potential cyclic voltammetry range. Experimental results showed ascorbic acid, dopamine, VB_1_, VB_6_, VB_2_, and uric acid etc don't interference with the NA assay, as shown in Table 1. Isonicotinic acid gives an oxidation peak at around 350 mV, which overlaps with the NA oxidation peak (320 mV), and thus interferes with the NA detection.

The developed CLTNPE was challenged by detect NA in human urine samples. Some NA was added to human urine, but the concentration of NA in human urine is in detection range (Figure 9). The samples were prepared according to the following procedure: Fifty milliliters of human urine were removed and mixed with 50 μL of 0.01 mol·L^-1^ NA solution and subsequently added with 0.295 g KCl to keep human urine contain 0·1 mol.L^-1^ KCl.

1.0×10 mol·L^-1^ NA was added to the urine sample. The preparation of other samples was similar to above process. In order to obtain the appropriate conditions, the pH of human urine sample solution was kept around 10. Five human urine sample solutions containing the different concentrations of NA were made basic using 0.5 mol·L^-1^ NaOH. Using the calibration curve obtained under optimal conditions, satisfactory recoveries were obtained, as shown in Table 2.

## 3. Experimental Section

SEM images of CLTN nanocomposite were obtained with a JSM-5600LV SEM (JEOL, Ltd., Japan). All cyclic voltammograms and chronoamperometry datas were acquired using a computer-based potentiostate/galvanostate (model 283, EG&GP Princeton Applied Research, Princeton, NJ, USA). The three-electrode system consists of a CNLTPE, a saturated calomel reference electrode (CE) and a platinum wire as an auxiliary electrode.

Multi-walled carbon nanotubes with ∼ 95% purity were kindly provided by the College of Materials Science and Engineering, Hunan University. Vitamin pp was purchased from Sigma (USA) and other chemicals were of analytical reagent grade. All solutions were prepared with double distilled water.

The La-TiO_2_ was prepared according to Peng's report [25]. Briefly, Ti(SO4)_2_ (1.1402 g) was dissolved in distilled water (3.5 mL). The resulting solution was added to cetyltrimethylammonium bromide (CTAB) solution under stirring with a final molar ratio of 0.95:0.05:0.12:100 (Ti(SO_4_)_2_:La(NO_3_)3:CTAB:H_2_O). The pH of mixture solution was adjusted to 0.6. After stirring for 30 min, the solution was aged at room temperature for 12 h, and then transferred into an autoclave and which was kept at 100°C for hydrothermal treatment. After 72 h, the resulting powders were cooled to room temperature, then recovered by centrifugation, washed with water and ethanol then dried at 120 °C overnight. In order to remove organic materials, ion-exchange treatment was performed by mixing the as-synthesized powders with a water and ethanol (molar ratio 1:1) solution of sodium chloride under stirring at 313 K for 5 h. The resulting solids were washed with water and ethanol then dried at 120°C overnight. The prepared samples were calcined at different temperatures 2 h to improve crystallinity with a heating rate of 2°C/min.

The preparation of CLTN included two steps. The first step was the preparation of oxidized carbon nanotubes (CNTs) with concentrated acid [26-27]. This process introduced oxygen-containing groups such as ether or carboxyl groups on the CNT surface and the long CNTs were cut into short ones. The oxidized CNTs (o-CNT) were washed with distilled water and dried at 100°C. The o-CNT (0.487 g) was mixed with water (10 mL) and the pH was adjusted to 11∼12 using NaOH solution. La-TiO_2_ (around 0.081 g) was added into the above suspension. The o-CNT and La-TiO_2_ mixture was sonicated for 2 hours and then dried at 100 °C overnight.

CLTNPE was prepared according to the procedure reported elsewhere [28] with minor modifications. In a typical process, paraffin (112.3 mg) was dissolved in ether and dried in air about 10 min. After evaporation of the excess of ether, the paraffin was mixed with an appropriate amount (210.2 mg) of CLTN. The resulting paste was squeezed into a PVC tube of 6 mm id to a depth of 1 cm. Inside the tube the mass was in contact with a conducting graphite rod, which was in turn connected with an electric wire to complete the measurement circuit. The preparation process of carbon paste electrode is similar to that of the CLTNPE, only with CLTN replaced by graphite powder.

Before using CLTNPE, GC and CPE, they were cleaned. A bare glassy carbon electrode was pretreated according to the following procedure. It was carefully polished with alumina until a mirror finish was obtained, after rinsing with the doubly distilled water to remove the alumina residues, the electrode was subsequently sonicated with 1+1 HNO_3_, ethanol and doubly distilled water for 10min,respectively, followed by thoroughly rinsing with the doubly distilled water. The preparation process is as follows: CLTNPE and CPE were polished with filter paper and rinsed with doubly distilled water.

## 4. Conclusions

The present study investigated the microstructure and electrochemical activity of CLTN in detail. The SEM measurements indicate the formation of the CLTN materials with microstructure which La-TiO_2_ tightly clings to o-CNT. The CLTNPE described in this paper provides a sensitive tool for investigating the electrochemistry of small molecules, such as potassium ferricyanide and ascorbic acid, and it can be used as an NA sensor. Compared with conventional carbon electrodes, such as CPE and GC, the proposed CLTNPE has better electron-transfer properties and the reversibility of the electrochemical reactions involving small molecules could be improved.

Without a mediator, CLTNPE as an NA sensor possesses high electrochemical activity and high affinity for NA. Other similar vitamins do not interfere with the NA detection. Under optimized experimental conditions, broad linear range and low detection limit were obtained. On the other hand, its ease of fabrication, nice stability and reproducibility are obvious advantages.

Using the human urine which is from my group as substrate solution, some NA was added to human urine, but the concentration of NA in human urine is within the detection range. The recovery of NA was monitored using the proposed CLTNPE. The mean recovery was 101.8%. The results of the determination of NA in human urine samples are satisfactory.

## Figures and Tables

**Table 1.** Interference chemicals and the results of interference level

**Interfering chemicals**	**Interference level**
VB_1_	No interference
VB_2_	No interference
VB_6_	No interference
L-cysteine	No interference
Ascorbic acid	Too low
Uric acid	No interference
H_2_O_2_	Too low
Dopamine	No interference
Isonicotinic acid	High

**Table 2.** Recovery of NA in human urine sample solution

**NA added (μmol L^-1^)**	**NA found (μmol·L^-1^)**	**RSD (%)**	**Recovery (%)**	**Mean (%)**
10.00	9.832	3.4	98.3	101.8
40.00	41.03	2.1	102.6	
50.00	52.23	2.7	104.5	
70.00	69.42	1.9	99.2	
90.00	93.78	2.9	104.2	
